# Plasmon-assisted trapping of nanoparticles using a silver-nanowire-embedded PMMA nanofiber

**DOI:** 10.1038/srep20433

**Published:** 2016-02-04

**Authors:** Chang Cheng, Xiaohao Xu, Hongxiang Lei, Baojun Li

**Affiliations:** 1 State Key Laboratory of Optoelectronic Materials and Technologies, School of Physics and Engineering, Sun Yat-Sen University, Guangzhou 510275, China

## Abstract

The integration of surface plasmon with waveguide is a strategy for lab-on-a-chip compatible optical trapping. Here, we report a method for trapping of nanoparticles using a silver nanowire (AgNW) embedded poly(methyl methacrylate) (PMMA) nanofiber with the assistance of surface plasmon polaritons (SPPs). The nanoparticles (polystyrene, 700 nm diameter) are transported along the nanofiber and ultimately trapped at the AgNW embedded region because of the enhanced optical gradient force towards the nanofiber exerted on the nanoparticles and optical potential well generated by the excitation of SPPs. The low optical power requirement and the easy fabrication of the AgNW-embedded nanofiber with broad range of wavelength for SPPs are advantageous to the applications in optofluidics and plasmofluidics.

Optical trapping techniques have been extensively developed as powerful tools for optical manipulation of micro-/nanoparticles with a broad range of applications such as chemical analysis^1^, optical sorting^2^ and biophotonic waveguides^3^. Among the various techniques^4^, evanescent-field based methods, such as slot waveguide^5^, optical fiber^6^,^7^, and photonic crystal resonators^8^ have been a promising choice because the exponentially-decaying evanescent field in cross section can overcome the diffraction limit to trap nanoparticles and the long propagating distance of the evanescent field can extend the trapping range. Additionally, the evanescent-field based methods can facilitate near-field coupling^9^ and excitation of internal resonances of the particles^6^,^10^, both plasmonic and optical ones. To enhance the strength of evanescent fields, confine the light more tightly and reduce the required optical power for optical trapping, surface plasmons—collective oscillation of free electrons confined tightly at the metal-dielectric interface including surface plasmon polaritons (SPPs) and localized surface plasmons (LSPs)—provide a solution by integrating metal structures to photonic devices^4^,^11^,^12^,^13^. For examples, gold stripe fabricated on the surface of Kretschmann prisms has been used to trap dielectric nanoparticles^14^, with the assistance of SPPs excited by the evanescent field outside the prism. The stripe reduces the usage of metal, so the absorption of light and thermal effect is minimized. Moreover, by fabricating gold disks array on the surface of dielectric waveguide^15^, the trapping of dielectric microparticles and yeast cells was realized based on SPPs, which demonstrates for the first time the applicability of plasmonic traps to lab-on-a-chip devices. After that, it has been demonstrated that gold nanospheres transported along an optical fiber can be accelerated by depositing gold nanoparticles on the surface of the optical fiber, owing to the LSPs supported by the gold nanoparticles^16^. However, these methods need complicated processes (e.g. electron-beam lithography) to integrate the metal structures with the photonic devices and the metal structures may be polluted due to the exposure to the circumstance. In addition, the LSP-based trapping needs specified resonant wavelengths to excite the surface plasmons. In this work, we report a plasmon-assisted trapping of nanoparticles by embedding a silver nanowire (AgNW) into a poly(methyl methacrylate) nanofiber (PMMA NF). Here, the PMMA nanofiber exhibits high optical transparency (92%), high flexibility, biocompatibility and good processability^17^. The AgNW can generate the enhanced optical gradient force and an optical potential well to stably trap the nanoparticles with the assistance of SPPs, thus the required optical power can be reduced. Also, the SPPs can be excited by a broad range of wavelengths^18^, that is, the plasmon-assisted trapping wavelength is nonselective. The AgNW-embedded nanofiber is easy to be fabricated by a direct drawing method and the PMMA cladding can prevent the possible pollution of AgNW. The thermal effect caused by the absorption of light by the AgNW is extremely small, which is beneficial for the plasmonic trapping.

## Results

### Plasmon-assisted trapping of nanoparticles

**Figure 1:**
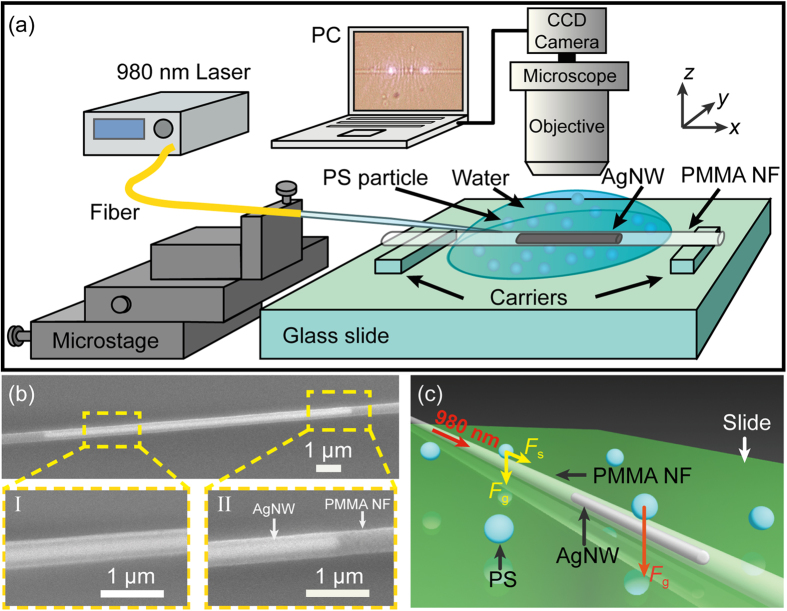
Schematic of the experimental setup. (**a**) Schematic illustration of the experimental setup. (**b**) SEM image of an AgNW-embedded PMMA nanofiber (NF). Insets I and II are the close-up views of the nanofiber. (**c**) Schematic of the plasmon-assisted trapping process of nanoparticles.

Figure 1a schematically presents the experimental setup. A droplet of 700 nm polystyrene (PS) nanoparticles suspension was injected on a clean glass slide by a micro-syringe. The AgNW-embedded PMMA nanofiber was immersed in the suspension with a 100 nm gap between the nanofiber and the glass slide using two carriers. Since the refractive index of the PMMA nanofiber (*n*_p_ = 1.49) is larger than that of the glass slide (*n*_g_ = 1.45), the gap can avoid the light leakage from the nanofiber to the glass slide. A laser beam at a wavelength of 980 nm was evanescently coupled to the nanofiber by an optical fiber taper (see Fig. S1 in Supplementary Information) fabricated using a flame-heating method. A computer interfaced microscope with a CCD camera is used to observe and acquire the dynamics of the PS nanoparticles. Figure 1b shows the scanning electron microscope (SEM) images of the AgNW-embedded PMMA nanofiber (average diameter: 400 nm). The diameter and length of the embedded AgNW are 220 nm and 12.6 μm, respectively. The close-up views of the nanofiber (insets I and II of Fig. 1b) indicate that it exhibits a smooth surface morphology and that the AgNW was embedded in the middle of the nanofiber. Figure 1c schematically shows the trapping process. The evanescent field propagating outside the nanofiber will produce an optical gradient force *F*_g_ in *y* direction, and thus draw the nanoparticles nearby towards the nanofiber surface. Meanwhile, an optical scattering force *F*_s_ in the direction of light propagation propels the nanoparticles along the nanofiber until to the AgNW embedded region, where the SPPs lead to an enhanced gradient force in *y* direction (see Simulation). Due to the finite length of the AgNW, the light field also generates an optical gradient and thus gradient force in *x* direction. Therefore, the resultant of scattering force and gradient force in *x* direction affects the dynamics of nanoparticle. The resultant force in *x* direction will decrease to a negative value at the AgNW embedded region, which generates an optical potential well. Ultimately, under the action of the enhanced gradient force in *y* direction and the optical potential well in *x* direction, the nanoparticle will be trapped stably in the AgNW embedded region.

**Figure 2:**
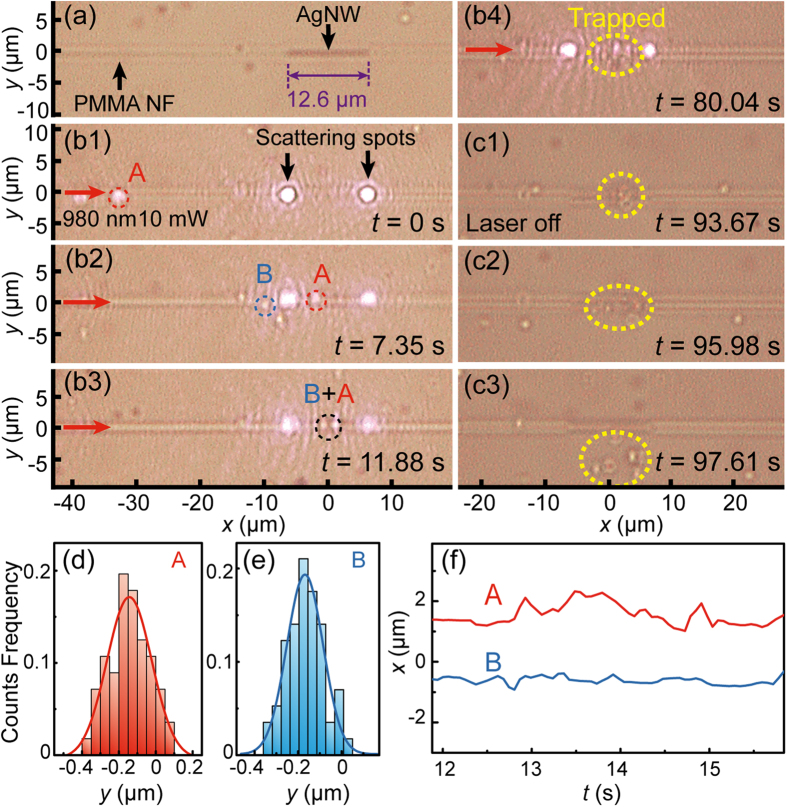
Plasmon-assisted trapping for 700 nm PS nanoparticles. (**a**) Optical microscope image of the AgNW-embedded PMMA nanofiber immersed in the PS nanoparticles suspension. (**b1–b4**) Plasmon-assisted trapping of nanoparitcles A and B. Input optical power of 980 nm laser is 10 mW. (**c1–c3**) Releasing of the trapped nanoparticles with the laser turned off. (**d,e**) Histograms and the corresponding fitted Gaussian curves of nanoparticles A and B’s positions in *y* direction from *t* = 11.88 to 15.84 s. (**f**) Positions of the two nanoparticles in *x* direction.

Figure 2a presents an optical microscope image of the AgNW-embedded PMMA nanofiber immersed in the PS nanoparticles suspension. Figures 2b1-b4 show the consecutive images for trapping of the nanoparticles. By launching the 980 nm laser with an optical power of 10 mW into the PMMA nanofiber (*t* = 0, Fig. 2b1), a nanoparticle (A) was attracted to the nanofiber due to the gradient force and then propelled in the direction of light propagation by the scattering force. It should be noted that, two scattering spots at the two ends of the AgNW were observed, which enabled the excitation of SPPs along the AgNW surface. At *t* = 7.35 s, the nanoparticle A was transported with a distance of 30.9 μm while a second nanoparticle (B) was attracted to the nanofiber and began to be propelled (Fig. 2b2). After another 4.53 s (i.e. *t* = 11.88 s, Fig. 2b3), both the nanoparticles A and B were trapped at the AgNW embedded region, which indicates that there is an optical potential well at the AgNW embedded region due to the excited SPPs. The detailed trapping process from *t* = 0 to 12 s is shown by Movie S1 in the Supplementary Information. When *t* = 80.04 s, more PS (~6) nanoparticles were transported and ultimately trapped at the AgNW embedded region, as shown in Fig. 2b4. At *t* = 93.67 s, the laser beam was turned off (Fig. 2c1) and the trapped nanoparticles began to diffuse in the water as a result of Brownian motion (Fig. 2c2,c3). The detailed releasing process from *t* = 88 to 98 s is shown by Movie S2 in the Supplementary Information. Therefore, it has been demonstrated that nanoparticles can be trapped and released with the assistance of the SPPs on the AgNW surface.

To know the trapping stiffness, the positions of the trapped nanoparticles A and B were analyzed from *t* = 11.88 to 15.84 s. In *y* direction, the histograms of A and B’s positions are shown in Fig. 2d,2e, which exhibit Gaussian distributions. The standard position deviations of the particles A and B in *y* direction are 0.11 and 0.07 μm, respectively. From the fitted Gaussian curves, the trapping stiffness *k* can be determined according to the equipartition theorem which can be expressed as^14^:


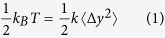


where *k*_*B*_ is the Boltzmann constant, *T* is the absolute temperature, and 〈Δ*y*^2^〉 is the positional variance of the trapped nanoparticle. According to Eq. (1), the trapping stiffness in *y* direction are calculated to be 0.37 and 0.75 pN/μm for the nanoparticles A and B, respectively, which demonstrates that the nanoparticles can be trapped steadily^14^. Figure 2f shows the positions of the two nanoparticles in *x* direction from *t* = 11.88 to 15.84 s. The standard position deviations of the particles A and B in *x* direction are 0.35 and 0.13 μm, respectively, and thus they can be trapped stably at the AgNW embedded region. Moreover, the vibration of the nanoparticle B is smaller than that of the nanoparticle A, which is owing to the larger trapping stiffness of the nanoparticle B compared with that of the nanoparticle A in *y* direction. In addition, it should be pointed out that the thermal effect^19^ due to the absorption of the light by the AgNW in the experiment was extremely small (see Fig. S2 in Supplementary Information), which is beneficial for the trapping of nanoparticles.

### Simulation and calculation

To explain the mechanism of the plasmon-assisted trapping of the nanoparticle at the AgNW embedded region, numerical analysis was performed based on the three-dimensional (3D) finite-difference time-domain (FDTD) method. The parameters are set to match the experimental conditions. The input power of the 980 nm laser is normalized to 1 W. The refractive indices of the PMMA nanofiber, PS nanoparticle and water are set as 1.49, 1.57 and 1.33, respectively. The refractive index of the AgNW is taken from Johnson and Christy^20^.

**Figure 3:**
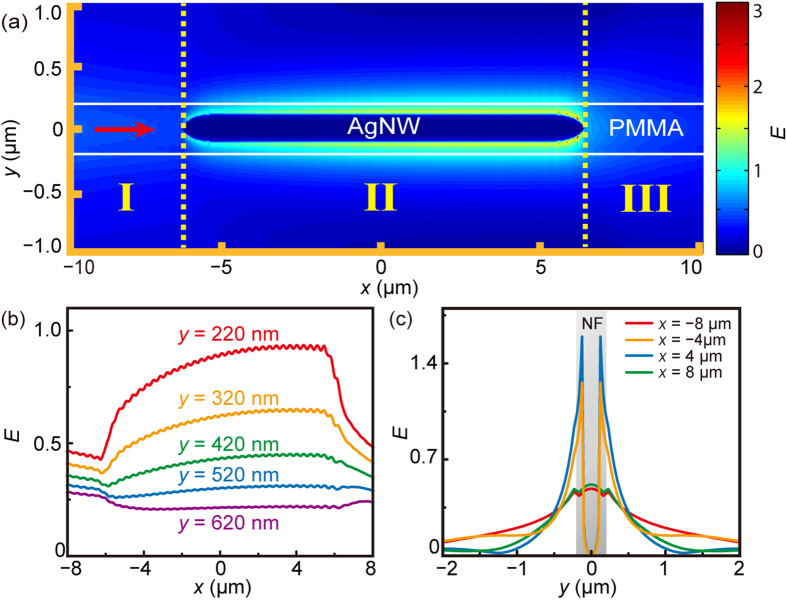
Electric field distribution (normalized to the input E-field) simulated by the 3D FDTD method. (**a**) Simulated E-field distribution. The regions I, II, III are the input region, AgNW embedded region and output region, respectively. (**b**) Distributions of normalized E-field in *x* direction at *y* = 220, 320, 420, 520 and 620 nm. (**c**) Distributions of normalized E-field in *y* direction at *x* = −8, −4, 4 and 8 μm.

Figure 3a shows the distribution of electric-field (E-field) amplitude normalized to the input E-field amplitude. It can be seen that, in the input region I (from *x* = −10 to −6.3 μm), a proportion of the laser propagates as evanescent wave outside the nanofiber. When light propagates to the AgNW embedded region II (from *x* = −6.3 to 6.3 μm), SPPs are excited on the surface of the AgNW, and hence the E-field around the AgNW is enhanced. As a result, the evanescent field outside the nanofiber is enhanced as well. After that, the E-field is decreased because no SPPs exist in the output region III (from *x* = 6.3 to 10 μm).

To further investigate the enhancement of E-field distribution, the distributions of the normalized E-fields in *x* direction with different *y* are plotted in Fig. 3b. It can be seen that due to the SPPs excited by the input light, the E-field around the nanofiber surface is enhanced in the AgNW embedded region. For instance, at *y* = 220 nm, the E-field in the AgNW embedded region is enhanced to ~2 times as that in the input region. With the increasing of *y*, however, the enhancement of E-field is decreasing. At *y* = 620 nm, the E-field in the AgNW embedded region is even weaker than that in the input region, which implies that the light is confined more tightly in the AgNW embedded region. The confinement of light can be characterized by the penetration depth of the evanescent field, which is defined as the depth at which the E-field falls to 1/e of its value at the nanofiber surface. As an example, based on the distributions of E-filed in *y* direction with *x* = −8, −4, 4 and 8 μm (Fig. 3c), the penetration depths are calculated to be 1.09, 0.27, 0.27 and 0.57 μm, respectively. The smaller penetration depths in the AgNW embedded region demonstrates that the light is confined more tightly in this region. Therefore, with the assistance of SPPs, the light field is enhanced and tightly confined, resulting a larger optical gradient and gradient forces exerted on nanoparticles as well in *y* direction, which can assist the trapping of nanoparticles.

**Figure 4:**
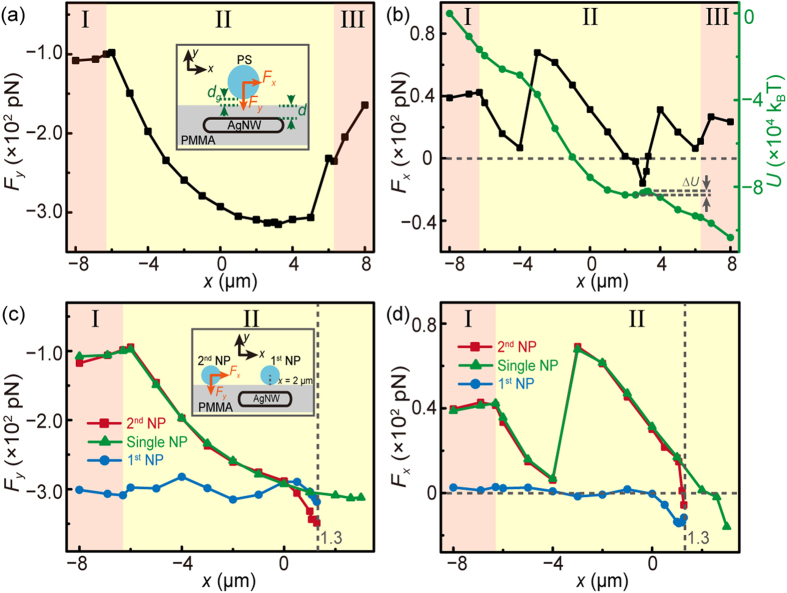
Calculated optical forces and optical potentials *U*. (**a**) Optical force in *y* direction (*F*_*y*_) exerted on a single PS nanoparticle as a function of *x*. The inset is the schematic illustration of the simulation. The gap between the nanofiber and PS (*d*_g_) is set to be 20 nm. The distance between the surfaces of AgNW and nanofiber (*d*) is set to be 90 nm. (**b**) Optical force in *x* direction (*F*_*x*_) and the optical potential as a function of *x*. (**c,d**) *F*_*y*_ and *F*_*x*_ exerted on the subsequent nanoparticle (2^nd^ NP) with a trapped nanoparticle (1^st^ NP) at *x* = 2 μm (red curves). The blue curves are *F*_*y*_ and *F*_*x*_ on the 1^st^ nanoparticle as a function of the 2^nd^ nanoparticle’s position. The green curves are the *F*_*y*_ and *F*_*x*_ on a single nanoparticle as in panel (**a**) and (**b**).

To explain the mechanism of the plasmon-assisted trapping of nanoparticles at the AgNW embedded region, optical forces exerted on a single nanoparticle are studied by integrating Maxwell stress tensor over a closed surface surrounding the nanoparticle. The gap between the nanoparticle and the nanofiber surface is set to be 20 nm as Debye length^21^ in the simulations (*d*_g_, inset of Fig. 4a), which plays a role on the optical force exerted on the nanoparticle (see Fig. S3 in Supplementary Information). The distance between the surfaces of the AgNW and the nanofiber (*d*) is set to be 90 nm to match the experiment conditions. The dependence of *F*_*y*_ on *x* is shown in Fig. 4a. Here, *F*_*y*_ is set to have the same positive direction as of *y* coordinate. It can be seen that *F*_*y*_ is negative, which keeps the nanoparticle attracted closely on the nanofiber surface. Meanwhile, owing to the enhancement of light supported by the SPPs, |*F*_*y*_| is increased in the AgNW embedded region. For example, *F*_*y*_ is increased to −312.1 pN at *x* = 3 μm, which is 2.9 times as that at *x* = −8 μm (|*F*_*y*_| = 107.7 pN). As for *F*_*x*_ (Fig. 4b), the increment in the AgNW embedded region is not obvious. However, it is interesting to find that *F*_*x*_ is turned to negative from *x* ≈ 2.0 to 3.3 μm, which implies that an optical potential well is generated. The green curve in Fig. 4b is the curve of optical potential (*U*) as a function of *x*, setting the zero point of the optical potential at *x* = –8 μm, from which the optical potential well depth (Δ*U*) is calculated to be 1.6 × 10^3^ *k*_*B*_·*T*. Therefore, under the action of the enhanced *F*_*y*_ and the optical potential well generated by SPPs at the AgNW the nanoparticles can be trapped at the AgNW embedded region.

In addition, a trapped nanoparticle has an influence on the optical force exerted on a subsequent nanoparticle^22^. To investigate this influence, the optical force exerted on the subsequent nanoparticle (2^nd^ NP) has been simulated with the trapped nanoparticle (1^st^ NP) at *x* = 2 μm (Fig. 4c,d). In Fig. 4c, *F*_*y*_ acting on the 2^nd^ nanoparticle (red curve) is not influenced when it is far from the 1^st^ nanoparticle (*x* <0 μm). But when the 2^nd^ nanoparticle is propelled close to the 1^st^ nanoparticle (*x* >0 μm), |*F*_*y*_| exerted on the 2^nd^ nanoparticle will increase more rapidly and become larger than that of a single nanoparticle (green curve). The larger *F*_*y*_ for the 2^nd^ nanoparticle implies that the trapping stiffness of the 2^nd^ nanoparticle will be stronger than that of the 1^st^ nanoparticle, which was demonstrated by the further theoretical calculation of the trapping stiffness for the two nanoparticles (see Fig. S4 in Supplementary Information). This conclusion can also explain why the trapping stiffness of the nanoparticle B (corresponding to the 2^nd^ nanoparticle in simulation) is stronger than that of the A (corresponding to the 1^st^ nanoparticle) in the above experiment. In addition, *d*_g_ is also a key factor. In the simulation, *d*_g_ is set to be 20 nm, but it has the possibility that the actual *d*_g_ for the nanoparticle B is smaller than that for the nanoparticle A in experiment, which will further increase the difference of the trapping stiffness between the two nanoparticles. In Fig. 4d, *F*_*x*_ on the 2^nd^ nanoparticle begins to decrease more rapidly after *x* = 1.07 μm. Therefore, the 2^nd^ nanoparticle will be slowed down and trapped before the 1^st^ nanoparticle. For the 1^st^ nanoparticle, when the 2^nd^ nanoparticle is close to it (*x* > 0 μm), a negative *F*_*x*_ will act on it (blue curve) and thus, the 1^st^ nanoparticle will be pulled in −*x* direction, which can explain the trapping position in experiment is a little off from the calculated one in Fig. 4b. Additionally, the viscous force of water will also lead to the shift of trapping position.

## Discussion

**Figure 5:**
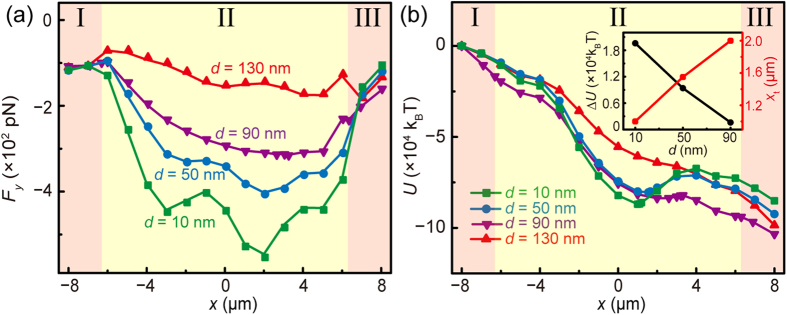
Calculated optical forces and optical potentials *U* for different *d*. (**a**) *F*_*y*_ and (**b**) optical potential *U* as a function of *x* for different *d*. The inset of (**b**) shows the dependence of potential well depth Δ*U* and trapping position *x*_t_ on *d*.

As the SPPs are confined tightly around the surface of the AgNW, the distance *d* between the surfaces of the AgNW and the nanofiber is influential to the evanescent field outside the nanofiber and thus the optical forces exerted on the nanoparticles. To know the effects of *d* on *F*_*y*_ and the optical potentials, further simulations were performed. Figure 5a shows the *F*_*y*_-*x* curves with *d* = 10, 50, 90 and 130 nm. It can be seen that |*F*_*y*_| in the AgNW embedded region is increasing with a decreasing *d*. For example, the maximum |*F*_*y*_| in the AgNW embedded region with *d* = 10 nm is 545.8 pN, which is 5 times as that in the input region (~110.0 pN), while the |*F*_*y*_| in the AgNW embedded region with *d* = 130 nm is barely enhanced compared with that in the input region. This is mainly because, for smaller *d*, more light enhanced by SPPs can be permeated outside the nanofiber surface. The optical potentials *U* along the nanofiber are also calculated with different *d* (Fig. 5b). It can be seen that, with an increasing *d*, the depth of optical potential well (Δ*U*) is decreasing and the trapping position (*x*_t_) is increasing (inset of Fig. 5b). For example, for *d* = 10 nm, Δ*U* = 2.0 × 10^4^ *k*_*B*_*·T* and *x*_t_ = 1.0 μm while for *d* = 90 nm, Δ*U* = 1.6 × 10^3^ *k*_*B*_*·T* and *x*_t_ = 2.0 μm. Especially, for *d* = 130 nm, no potential well exists and thus the trapping of nanoparticles cannot be realized. This is mainly because, for a larger *d*, the influence of SPPs on the nanoparticle is weaker. Therefore, the trapping performance can be further improved by embedding the AgNW nearer to the PMMA nanofiber surface.

In conclusion, a simple and compact strategy for realizing optical trapping of nanoparticles is proposed and demonstrated by embedding a silver nanowire (AgNW) into a PMMA nanofiber with the assistance of surface plasmon polaritons (SPPs). Owing to the excitation of SPPs on the AgNW surface, the evanescent field is enhanced and tightly confined, leading to an enhanced optical gradient force towards the nanofiber and an optical potential well along the nanofiber to trap the nanoparticle. Further calculations show that the trapping performance can be improved by embedding the AgNW closer to the nanofiber surface. Owing to the advantages of low optical power requirement, easy fabrication and broad range of wavelength for SPPs, we anticipate this technique will bring potential applications in chemical/biological analysis, directed assembly of nanomaterials and targeted particle/drug delivery in lab-on-a-chip systems.

## Methods

### Fabrication of AgNW-embedded PMMA nanofiber

The AgNW-embedded PMMA nanofiber was fabricated by a direct drawing method from PMMA solutions with doped AgNWs. The fabrication details were as follows: First, AgNWs ethanol solution (XFNANO Inc., China) were dried at room temperature for 10 min and then dispersed in 2 ml toluene solution to obtain 1% (w/v) AgNWs toluene solution with an assistance of ultrasonication. After that, 0.2 g PMMA was dissolved in the AgNWs toluene solution and stirred magnetically for 10 h to obtain a solution mixture with an appropriate viscosity for drawing of nanofiber. Then, a silica fiber taper tip (diameter ~1 μm) was immersed into the solution for 1–2 s and dragged out with a speed of 0.1–1 m/s, leaving an AgNW-embedded PMMA nanofiber with a diameter of 400 ± 30 nm extending between the solution and the fiber taper. As the toluene evaporated quickly, an AgNW-embedded PMMA nanofiber was fabricated. The orientation of the AgNW is parallel to the long axis of the nanofiber, because of the strong shear forces exerted on the AgNW during the drawing process^23^. The nanofiber is of high diameter uniformity (see Fig. S5 in Supplementary Information) and the typical length can reach to several centimeters.

### Preparation of PS nanoparticles suspension

The PS nanoparticles were diluted in deionized water with with assistance of ultrasonication (volume ration of PS nanoparticles to water is about 1:1000). After that, a droplet of the PS suspension was injected onto the surface of a glass slide with a micro-syringe.

### Measurement of particle positions

The particle positions were measured by the particle-tracking software^24^, and the error on position measurement is estimated to be 10 nm^25^. According to error accumulation, the error on the measurement of trapping stiffness is calculated to be ~0.01 pN/μm.

## Additional Information

**How to cite this article**: Cheng, C. *et al.* Plasmon-assisted trapping of nanoparticles using a silver-nanowire-embedded PMMA nanofiber. *Sci. Rep.* **6**, 20433; doi: 10.1038/srep20433 (2016).

## Supplementary Material

Supplementary Information

Supplementary Movie S1

Supplementary Movie S2
