# Supplementary material for: Plasmon-assisted trapping of nanoparticles using a silver-nanowire-embedded PMMA nanofiber
Source: Sci Rep. 2016 Feb 4;6:20433. doi: 10.1038/srep20433 (PMC4740807; doi:10.1038/srep20433)
Supplement: Supplementary Information [file srep20433-s1.pdf]

Supplementary Information

Plasmon-assisted trapping of nanoparticles using a silver-nanowire-embedded PMMA nanofiber

Chang Cheng, Xiaohao Xu, Hongxiang Lei*, and Baojun Li*
State Key Laboratory of Optoelectronic Materials and Technologies, School of Physics and Engineering, Sun Yat-Sen University, Guangzhou 510275, China
**leihx@mail.sysu.edu.cn, stslbj@mail.sysu.edu.cn*

1. Optical coupling between fiber taper and PMMA nanofiber

The laser is coupled to the PMMA nanofiber evanescently by an optical fiber taper. Figure S1 shows the schematic of the coupling structure. The optical fiber taper was moved with the assistance of the microstage and placed in close contact with the PMMA nanofiber in parallel, so optical field in the fiber taper and nanofiber can be strongly overlap, resulting in efficient coupling. The estimated coupling efficiency is 90%.

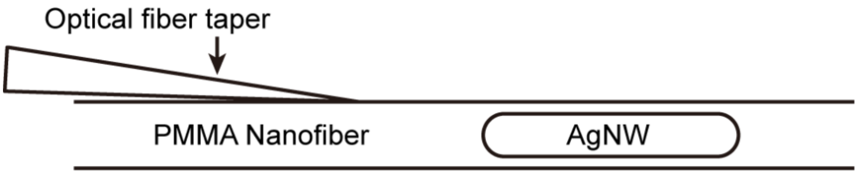

Figure S1. Schematic of the coupling structure.

2. Thermal effect

The temperature increment for AgNW with a length L and diameter D embedded in the PMMA nanofiber can be expressed as (Ref. 19)

$$\Delta T = \frac{P}{4\pi R_{\mathrm{eq}}\beta\kappa}, \tag{1}$$

where P is the total absorption power, R_{eq} is the radius of a sphere with same volume as the nanowire, β is the dimensionless thermal-capacitance coefficient, and κ is the thermal conductivity of the PMMA (~0.18 W $\cdot$m^{-1} K^{-1}). The variable P, R_{eq},and β can be expressed as

$$P = \frac{\omega}{2\pi}\mathrm{Im}(\varepsilon)\int|\mathbf{E}|^2\,\mathrm{d}\mathbf{r}, \tag{2}$$

$$R_{\mathrm{eq}} = [(3L–D)D^2/16]^{1/3}, \tag{3}$$

and

$$\beta = 1+0.96587\ln^2(L/D), \tag{4}$$

respectively, where $\omega/2\pi$ is the light frequency, Im(ε) is the imaginary part of the

permittivity of the AgNW, **E** is the electric field, which can be obtained by FDTD simulation. In this work, the total absorption power P is calculated to be 45 μW. For the AgNW with L = 12.6 μm and D = 220 nm used in the experiment, R_{eq} and β are calculated to be 0.48 μm and 16.83, respectively. Thus the temperature increment at the AgNW surface is ~2.4 K.

To get the temperature increment at the interface of the PMMA nanofiber and the water, the temperature distribution is simulated by COMSOL 4.3 (Fig. S2). The room temperature and the AgNW temperature are set to be 293.15 and 295.55 K, respectively. The curve in Fig. S2b shows the temperature distribution in z direction at x = 0 μm. It can be seen that the temperature at the PMMA-water interface is ~294.75 K, thus the temperature increment at the interface of the nanofiber and the water is ~1.6 K. Therefore, the thermal effect is extremely small and can be ignored.

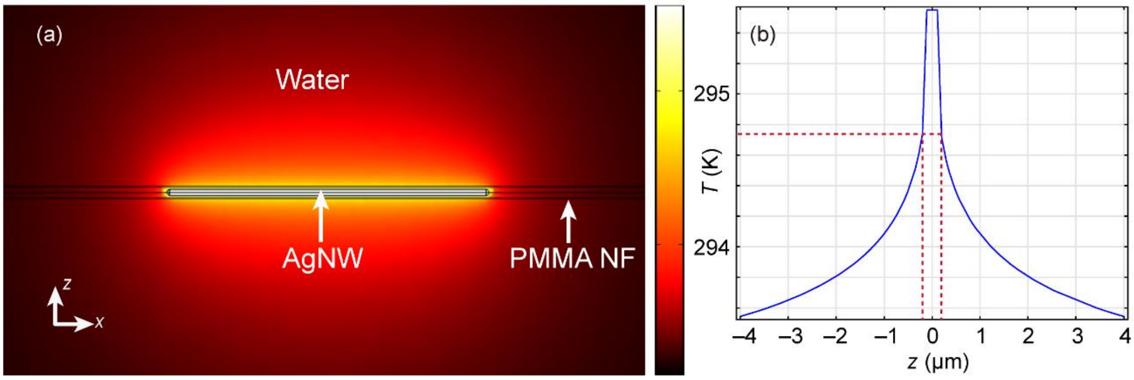

Figure S2. Temperature distribution for the AgNW-embedded nanofiber in water.

3. Dependence of optical forces on d_g

To know the influence of d_g between the nanoparticle and the nanofiber surface on the optical forces, simulations are performed with d_g to be 10, 20, and 30 nm, respectively, as shown in Fig. S3. It can be seen that, F_z is larger for smaller d_g (Fig. S3a), because the light field is stronger at the position close to the nanofiber surface. Meanwhile, F_x is almost the same for d_g = 10, 20, and 30 nm, with the negative values between ~2.0 to 3.3 μm (Fig. S3b), which means that the nanoparticle will be trapped at the same position.

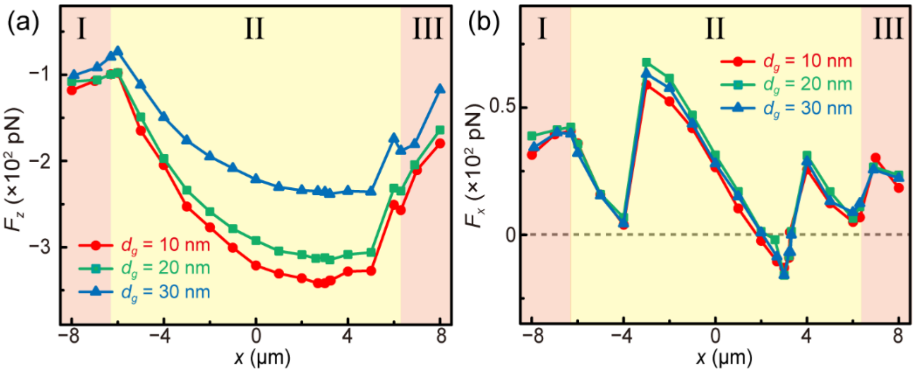

Figure S3. Optical forces exerted on the nanoparticle with different d_g.

4. Trapping stiffness

To theoretically predict the trapping stiffness and compare the predicted stiffness with the experiment value, the simulation and the related calculation have been performed. Figure S4 shows the calculated optical force in *y* direction (F_y) as a function of *y* for the 1st nanoparticle (trapped at x = 2 μm) and the 2nd nanoparticle (trapped at x = 1.2 μm), respectively. From the curves, the trapping stiffness in *y* direction is obtained to be 3.79 and 5.04 pN/μm for the 1st and 2nd nanoparticle, respectively by taking the experimental optical power of 10 mW into account. The predicted stiffness for the 2nd nanoparticle (corresponding to the nanoparticle B in the experiment) is stronger than that for the 1st nanoparticle (corresponding to the nanoparticle A), which is consistent with the experiment result. In addition, the predicted stiffness is stronger than the measured values, which is mainly caused by the perturbation of the circumstance in experiment, such as Brownian motion that is not taken into account in simulation.

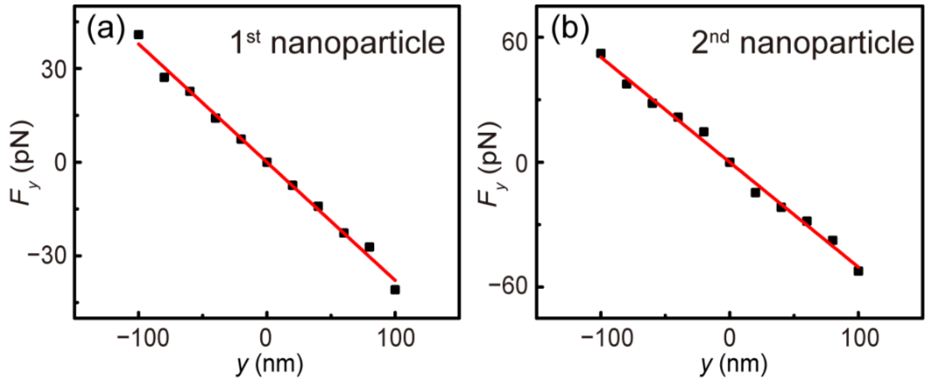

Figure S4. Optical force in *y* direction for the 1st and 2nd nanoparticles as a function of *y*.

5. Diameter uniformity of PMMA nanofiber

Take a typical AgNW-embedded PMMA nanofiber as an example (Figure S5), the average diameter *D* is ~390 nm and the length *L* is ~66 μm. The maximum diameter variation $\Delta D \approx 30$ nm, and the calculated diameter variation ration $\Delta D/L \approx 4.5\times10^{-4}$. Therefore, the fabricated AgNW-embedd PMMA nanofiber has high diameter uniformity.

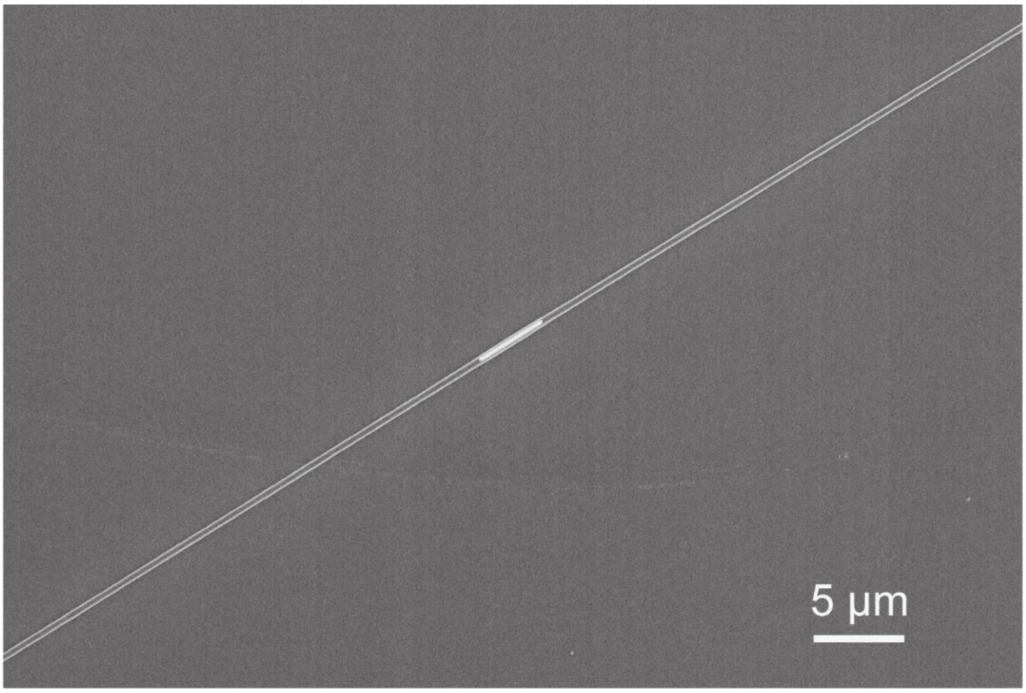

Figure S5. SEM image of a typical AgNW-embedded PMMA nanofiber.

6. Movies

Movie S1 shows the trapping process of nanoparticles A and B from t = 0 to 12 s. Movie S2 shows the trapping and releasing process of nanoparticles from t = 88 to 98 s.
